# Morphological and molecular identification of two new wood-inhabiting species of *Hyphoderma (Basidiomycota)* from Qinghai, northwestern China

**DOI:** 10.3897/mycokeys.141.210190

**Published:** 2026-10-09

**Authors:** Xin Li, Yujin Cui, Rui Xing, Yucheng Dai, Yuan Yuan, Yingda Wu

**Affiliations:** 1 State Key Laboratory of Efficient Production of Forest Resources, School of Ecology and Nature Conservation, Beijing Forestry University, Beijing 100083, China State Key Laboratory of Efficient Production of Forest Resources, School of Ecology and Nature Conservation, Beijing Forestry University Beijing China; 2 Northwest Institute of Plateau Biology, Chinese Academy of Sciences, Qinghai 810008, China Northwest Institute of Plateau Biology, Chinese Academy of Sciences Qinghai China; 3 Key Laboratory of Ministry of Emergency Management for Forest and Grassland Fire Risk Prevention, China Fire and Rescue Institute, Beijing 102202, China Key Laboratory of Ministry of Emergency Management for Forest and Grassland Fire Risk Prevention, China Fire and Rescue Institute Beijing China

**Keywords:** *
Hyphodermataceae
*, phylogeny, taxonomy, wood-rotting fungi

## Abstract

Wood-inhabiting fungi serve as primary decomposers of lignocellulose in forest ecosystems, facilitating carbon release and nutrient turnover. These processes are essential for sustaining material cycling, energy flow, and soil health. The genus *Hyphoderma*, within the *Basidiomycota*, is a notable group among wood-inhabiting fungi. This study introduces two new resupinate, adnate, wood-inhabiting fungal taxa, *Hyphoderma tenuicystidiatum* and *H. tabuliformis*, based on morphological and molecular phylogenetic analyses. *Hyphoderma tenuicystidiatum* is distinguished by light vinaceous gray fresh basidiomata with a smooth hymenophore, a monomitic hyphal system with clamp connections on generative hyphae, cylindrical cystidia, the presence of fusoid cystidioles, and subcylindrical to cylindrical basidiospores measuring 10.4–14.9 × 5.2–7.1 µm. In contrast, *H. tabuliformis* exhibits white to cream fresh basidiomata with an arachnoid hymenophore, encrusted generative hyphae, clavate cystidia, and subcylindrical basidiospores measuring 8.7–12.2 × 3.6–4.2 µm. The discovery of two new species in Qinghai enriches the diversity of *Hyphoderma* and provides additional morphological and molecular data for the genus.

## Introduction

Wood-inhabiting fungi grow on various woody materials and constitute an important group of microorganisms (Yuan et al. 2020, 2026; Wu et al. 2022; Liu et al. 2025; Guan et al. 2026). Some of these fungi can decompose and utilize wood, thereby facilitating nutrient cycling in forest ecosystems, and are vital components of these ecosystems (Si et al. 2011; Wang et al. 2023). In addition, certain species, such as medicinal fungi and forest pathogens, have significant economic value (Wu et al. 2019; Yuan et al. 2021; Bhunjun et al. 2024). Although several wood-inhabiting fungi have been described from western China (Dai et al. 2004a; Wu et al. 2017; Yuan et al. 2021), the diversity of this group remains poorly known. *Hyphoderma* Wallr., a member of the family *Hyphodermataceae* Jülich (*Polyporales*, *Basidiomycota*), was established by Wallroth (1833) and typified by *H. setigerum* (Fr.) Donk. The genus is characterized by resupinate to effuse-reflexed, adnate, whitish to yellowish basidiomata of ceraceous consistency, a smooth to tuberculate or hydnoid hymenophore, a monomitic (rarely dimitic) hyphal system with clamp connections, the presence or absence of cystidia, suburniform to subcylindrical basidia, and ellipsoid to subglobose, smooth, thin-walled basidiospores (Bernicchia and Gorjón 2010).

The genus *Hyphoderma* was first introduced by Wallroth (1833) with eight species based on a broad concept that included both resupinate and thelephoroid (clavarioid) forms. After its establishment, the genus was largely neglected until the mid-20^th^ century, when the Dutch mycologist M.A. Donk undertook extensive revisions (Donk 1949, 1957). Donk (1949) selected *Hyphoderma spiculosum* (syn. *Thelephora setigera*) as the type, arguing that it was the most prominent element among the taxa listed by Wallroth and also the one most in accordance with the genus description. Subsequently, Donk (1957) provided a more thorough revision of the genus, recognizing 25 species and formally adopting the combination *H. setigerum* (Fr.) Donk for the type species, thereby confirming *H. spiculosum* as a later synonym of *H. setigerum*. Following Donk’s revisions, several taxonomists attempted to subdivide the genus at the sectional level (Parmasto 1968; Eriksson and Ryvarden 1975).

Parmasto (1968) divided *Hyphoderma* into three sections, namely *Hyphoderma*, *Mutatoderma*, and *Stephanocystis*. Eriksson and Ryvarden (1975) considered the genus to be unclearly delimited and, anticipating a future subdivision, proposed 12 possible natural species groups as a basis for future taxonomic revision. In the 21^st^ century, molecular phylogenetic studies have profoundly reshaped the traditional classification of *Hyphoderma* (Larsson 2007). As a result, species possessing distinctive structures such as echinocysts were segregated and transferred to a reinstated older genus, *Peniophorella*. This changed the concept of *Hyphoderma* to a narrower circumscription.

To date, 137 species of *Hyphoderma* have been discovered worldwide, among which 45 have been reported from China (Guan and Zhao 2021; Li et al. 2026). During investigations of the diversity of wood-rotting fungi in China, four *Hyphoderma*-like samples were collected. Phylogenetic analyses based on nuclear ribosomal ITS and nLSU sequences were carried out to confirm their taxonomic status. Morphological and molecular evidence confirmed that the four examined specimens belong to two new *Hyphoderma* species.

## Materials and methods

### Morphological studies

The examined specimens are deposited in the Fungarium of the Institute of Microbiology, Beijing Forestry University (**BJFC**), Beijing, China. Macromorphological characteristics, including the color and thickness of the basidiomata, hymenophoral surface, and sterile margin, were examined under a stereomicroscope (Nikon SMZ 1000, Tokyo, Japan) at 4× magnification. Colors used follow Anonymous (1969) and Petersen (1996). Samples for microscopic examination were stained in Cotton Blue, Melzer’s reagent, 5% potassium hydroxide, and distilled water, and sections or tissues of basidiomata were studied in Cotton Blue at 1000× magnification using a Nikon E80i microscope with phase-contrast illumination (Nikon, Tokyo, Japan). Line drawings were made with the aid of a drawing tube. To show variation in spore sizes, 30 basidiospores were measured from each specimen. Stalks were excluded from basidia measurements, and the hilar appendage was excluded from basidiospore measurements (Yuan and Zhao 2024). Basidiospore measurements are presented as follows: (*c*) *d*–*e* (*f*) × (*g*) *h*–*i* (*j*) µm, where c and f represent the extreme minimum and maximum values of basidiospore length, each accounting for 5% of the measurements; d and e represent the minimum and maximum values within the 90% range; basidiospore width values (*g*–*j*) are given in the same manner. The following abbreviations were used: ***L*** = mean spore length (arithmetical average of all spores), ***W*** = mean spore width (arithmetical average of all spores), ***Q*** = values of the length/width ratios among the studied specimens, ***n*** = *a*/*b* (number of spores (*a*) measured from a given number (*b*) of specimens), **IKI** = Melzer’s reagent, **IKI−** = negative in Melzer’s reagent, **KOH** = 5% potassium hydroxide, **CB** = Cotton Blue, **CB−** = acyanophilous.

### DNA extraction, polymerase chain reaction amplification, and sequencing

A CTAB plant genome rapid extraction kit-DN14 (Aidlab Biotechnologies Co., Ltd.) was used for DNA extraction and PCR amplification from dried specimens according to the manufacturer’s instructions, with some modifications (Liu et al. 2023). The ITS region was amplified with the primers ITS5 (5'-GGAAGTAAAAGTCGTAACAAGG-3') and ITS4 (5'-TCCTCCGCTTATTGATATGC-3') (White et al. 1990). The nLSU gene was amplified with the primers LR0R (5'-ACCCGCTGAACTTAAGC-3') and LR7 (5'-TACTACCACCAAGATCT-3') (Vilgalys and Hester 1990). The PCR procedure for ITS was as follows: initial denaturation at 95 °C for 3 min, followed by 35 cycles at 94 °C for 40 s, 58 °C for 45 s, and 72 °C for 1 min, and a final extension at 72 °C for 10 min. The PCR procedure for nLSU was as follows: initial denaturation at 94 °C for 1 min, followed by 35 cycles at 94 °C for 30 s, 48 °C for 1 min, and 72 °C for 1.5 min, and a final extension at 72 °C for 10 min. The PCR products were purified and sequenced bidirectionally by Sanger sequencing using the same primers at the Beijing Genomics Institute, Beijing, China. The newly generated sequences were deposited in GenBank. All sequences analyzed in this study are listed in Table 1.

**Table 1.** Information on specimens and GenBank accessions used in this study. New sequences are given in bold. Type specimens are indicated with an asterisk, and “-” indicates unavailable data.

**Species**	**Specimen no**.	**Locality**	**GenBank accession no**.	
			**ITS**	**nLSU**
*Climacocystis borealis*	FD-31	USA	KP135308	KP135210
*Diplomitoporus crustulinus*	FD-137	USA	KP135299	KP135211
*Hyphoderma alboarachnum*	CLZhao 30488*	China	PV470563	–
*H. amoenum*	USO 286622	Canada	HE577030	–
*H. asianum*	CLZhao 18091*	China	OR141726	PP826262
*H. assimile*	CBS:125852	New Zealand	MH863808	MH875272
*H. australosetigerum*	MA Fungi 92235*	Chile	MN963764	–
*H. australosetigerum*	MA Fungi 92240	Chile	MN963760	–
*H. bambusinum*	CLZhao 29903*	China	PV469674	PV819428
*H. cinereofuscum*	CLZhao 30341*	China	PQ492371	PQ511128
*H. cinereofuscum*	CLZhao 30283	China	PQ492372	PQ511129
*H. cremeoalbum*	NH 11538 (GB)	Sweden	DQ677492	DQ677492
*H. crystallinum*	CLZhao 9338*	China	MW917161	MW913414
*H. crystallinum*	CLZhao 9374	China	MW917162	MW913415
*H. definitum*	NH 12266 (GB)	China	DQ677493	DQ677493
*H. fissuratum*	CLZhao 6731	China	MT791331	–
*H. fissuratum*	CLZhao 6726*	China	MT791330	MT791334
*H. floccosum*	CLZhao 17129*	China	MW301683	MW293733
*H. floccosum*	CLZhao 17215	China	MW301687	MW293735
*H. fulgens*	CLZhao 37429*	China	PV829544	PV810095
*H. fulgens*	CLZhao 39474	China	PV829547	PV810098
*H. granuliferum*	5273	Canada	JN710545	JN710545
*H. grandineum*	CLZhao 30046*	China	PV470561	PV819429
*H. grandineum*	CLZhao 43328	China	PV470562	PV819430
*H. guangdongense*	CLZhao 12657*	China	PP235513	PP235514
*H. laceratum*	CLZhao 34242*	China	PV829552	PV810101
*H. laceratum*	CLZhao 34672	China	PV829553	PV810103
*H. litschaueri*	NH 7603 (GB)	Sweden	DQ677496	DQ677496
*H. litschaueri*	FP-101740-Sp	USA	KP135295	KP135219
*H. macaronesicum*	MA Fungi 90388	USA	KC984327	–
*H. macaronesicum*	TFC Mic 15115	China	HE577011	–
*H. marginatum*	CLZhao 3404	China	OM985717	OM985754
*H. medioburiense*	NH 10950 (GB)	Spain	DQ677497	DQ677497
*H. membranaceum*	CLZhao 5844*	China	MW917167	MW913420
*H. membranaceum*	CLZhao 6971	China	MW917168	MW913421
*H. microporoides*	CLZhao 6857*	China	MW917169	MW913422
*H. microporoides*	CLZhao 8695	China	MW917170	MW913423
*H. moniliforme*	Wu 0211-42*	China	KC928282	–
*H. moniliforme*	Wu 0211-46	China	KC928284	–
*H. mopanshanense*	CLZhao 6498*	China	MT791329	MT791333
*H. mopanshanense*	CLZhao 6449	China	OM985720	OM985759
*H. nemorale*	TNM F3931	China	KJ885183	KJ885184
*H. nemorale*	Wu 9508-14*	China	KC928280	KC928281
*H. niveomarginatum*	CLZhao 25078*	China	OR141728	OR506179
*H. nudicephalum*	Wu9307_29	China	AJ534269	–
*H. nudicephalum*	CLZhao 17839	China	OM985721	OM985760
*H. obtusiforme*	KHL1464	Spain	JN572909	–
*H. obtusiforme*	KHL11105	Spain	JN572910	–
*H. occidentale*	KHL 8477 (GB)	China	DQ677499	DQ677499
*H. paramacaronesicum*	MA Fungi 87736*	USA	KC984399	–
*H. paramacaronesicum*	MA Fungi 87737	China	KC984405	–
*H. pinicola*	Wu 0108-32	China	KJ885181	KJ885182
*H. pinicola*	Wu 0108-36	China	KC928278	KC928279
*H. prosopidis*	ARIZ HHB 8479	China	HE577029	–
*H. puerense*	CLZhao 9476*	China	MW443045	–
*H. puerense*	CLZhao 9583	China	MW443046	MW443051
*H. qujingense*	CLZhao 26018*	China	OR141729	PP826263
*H. setigerum*	FCUG 1200	Sweden	AJ534273	–
*H. setigerum*	FCUG 1688	Sweden	AJ534272	–
*H. sinense*	CLZhao 7963	China	MW301679	MW293730
*H. sinense*	CLZhao 17811*	China	MW301682	MW293732
*H. sordidum*	CLZhao 27379	China	OR141731	–
*H. sordidum*	CLZhao 27390*	China	OR141732	OR506180
***H. tabuliformis***	Dai 35238*	**China**	**PZ745286**	**PZ745283**
***H. tabuliformis***	**Dai 35239**	**China**	**PZ745287**	–
***H. tenuicystidiatum***	**Dai 31215**	**China**	**PZ745288**	**PZ745284**
***H. tenuicystidiatum***	Dai 29223*	**China**	**PZ745289**	**PZ745285**
*H. tenuissimum*	CLZhao 7221*	China	MW443049	MW443054
*H. tenuissimum*	CLZhao 16210	China	MW443050	MW443055
*H. tongbiguanense*	CLZhao 39574	China	PV470531	–
*H. tongbiguanense*	CLZhao 39575	China	PV470532	–
*H. transiens*	NH 12304	Sweden	DQ677504	DQ677504
*H. tropicum*	CLZhao 17308*	China	OM985727	OM985768
*H. variolosum*	CBS 734.91	China	MH862320	MH873992
*H. variolosum*	CBS 735.91	China	MH862321	MH873993
*H. weishanense*	CLZhao 22403*	China	OR141727	OR506181
*H. yingjiangense*	CLZhao 37195	China	PV470533	–
*H. yingjiangense*	CLZhao 37232	China	PV470534	–
*H. yunnanense*	CLZhao 8845*	China	OM985728	OM985769

### Phylogenetic analyses

The phylogenetic relationships of *Hyphoderma* were analyzed based on the ITS + nLSU sequences. *Climacocystis borealis* (Fr.) Kotl. & Pouzar and *Diplomitoporus crustulinus* (Bres.) Domański were used as the outgroups. To determine the phylogenetic affinities of the new species, the ITS sequences of the holotype specimens were used as queries in BLAST searches against the GenBank nucleotide database (Sayers et al. 2024), and the top hits with the highest similarity scores were retrieved for comparative analysis. The DNA sequences were aligned using MAFFT v. 7 with the G-INS-i option (Katoh and Standley 2013). Alignments were optimized manually in BioEdit v. 7.0.9 (Hall 1999) and concatenated in Mesquite v. 3.2 (Maddison and Maddison 2017). The partition homogeneity test (PHT; Farris et al. 1994) for the dataset was conducted in PAUP v. 4.0b10 (Swofford 2002) using 1,000 homogeneity replicates. The combined dataset was initially divided into four data blocks (ITS1, 5.8S, ITS2, and nLSU). The optimal partition scheme and substitution models for Bayesian inference (BI) were selected using PartitionFinder (Lanfear et al. 2017; Zhang et al. 2020) under the Akaike information criterion (AIC) within PhyloSuite v. 1.2.3. For maximum likelihood (ML) analysis, the optimal substitution models were automatically determined by ModelFinder (Kalyaanamoorthy et al. 2017), as implemented in IQ-TREE v. 2.2.0 (Minh et al. 2020).

**Figure 1.**
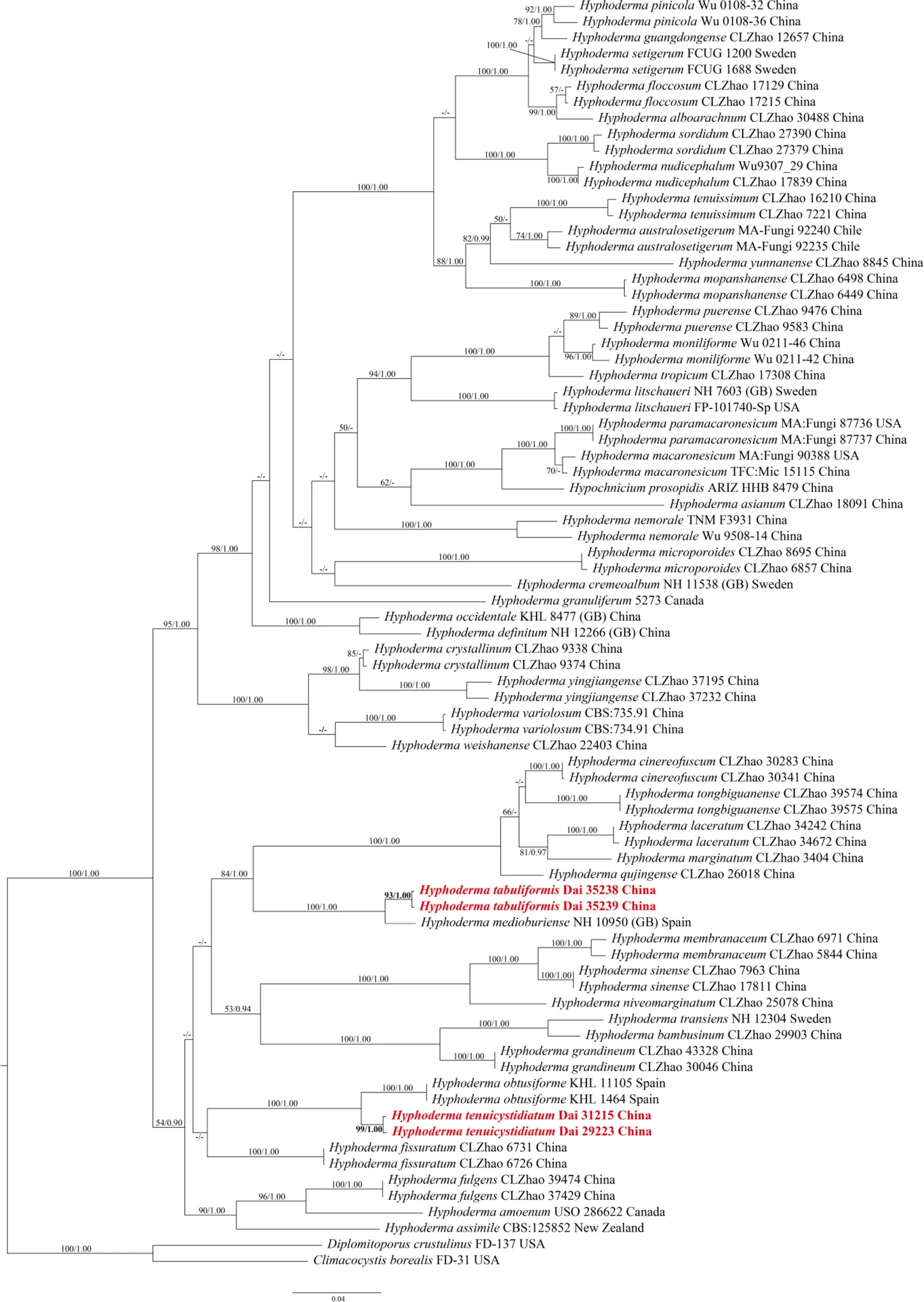
ML analysis of *Hyphoderma* based on the ITS + nLSU dataset. ML bootstrap values equal to or higher than 50% and Bayesian posterior probability values equal to or greater than 0.90 are shown. New taxa are given in bold.

The ML analyses were performed using IQ-TREE v. 2.2.0 (Minh et al. 2020) with the optimal substitution models determined by ModelFinder (Kalyaanamoorthy et al. 2017). Branch support was assessed using ultrafast bootstrap analysis with 5,000 replicates (Hoang et al. 2018). The BI analysis with MrBayes v. 3.2.7 (Ronquist et al. 2012) implemented the Markov chain Monte Carlo (MCMC) technique. Four Markov chains were run in two runs from random starting trees for 6 million generations for ITS + nLSU (Fig. 1), with one tree retained every 100^th^ generation until the average standard deviation of split frequencies was below 0.01. The first 25% of the sampled trees were discarded as burn-in, and the remaining trees were used to reconstruct a majority-rule consensus and to calculate Bayesian posterior probabilities (BPP) for the clades. The sequence alignments and the retrieved topologies were deposited in TreeBASE (http://www.treebase.org), under accession ID 32742. Phylogenetic trees were visualized with FigTree v. 1.4.2 (http://tree.bio.ed.ac.uk/software/figtree), and additional layout was carried out using Adobe Illustrator CS v. 5. Branches that received ML bootstrap support ≥ 50% and BPP ≥ 0.90 were considered significantly supported. ML bootstrap support ≥ 50% and BPP ≥ 0.90 are presented on the topologies from the ML analysis.

## Results

### Phylogenetic analyses

In this study, 134 sequences from 79 fungal samples representing 50 taxa were used to reconstruct phylogenetic trees, including 79 ITS sequences and 55 nLSU sequences. The dataset had an aligned length of 2,040 characters, of which 1,397 (68%) were constant, 116 (6%) were variable and parsimony-uninformative, and 527 (26%) were parsimony-informative. For the BI analysis, the combined dataset was partitioned into three subsets (ITS1 + ITS2, 5.8S, and nLSU) based on the PartitionFinder results. The best-fit models selected under the Akaike information criterion (AIC) were GTR+I+G for ITS1 + ITS2, K80+I+G for 5.8S, and GTR+I+G for nLSU. For the ML analysis in IQ-TREE, the dataset was partitioned into three subsets (ITS1, 5.8S, and ITS2 + nLSU), with the best-fit models being GTR+I+G for ITS1, K80+I+G for 5.8S, and GTR+I+G for ITS2 + nLSU. The phylogenetic tree topology from the Bayesian analysis was similar to that from the ML analysis, so only the ML tree is provided (Fig. 1).

### Taxonomy

#### Hyphoderma tabuliformis

Taxon classificationFungiPolyporalesHyphodermataceae

X. Li, Yuan Yuan & Y.C. Dai sp. nov.

27FBFE96-920F-5F97-9B39-E574A4C72B34

864738

Figs 2, 3, 4

##### Diagnosis.

*Hyphoderma tabuliformis* is distinguished by membranaceous to ceraceous basidiomata when fresh, arachnoid hymenial surface, clavate cystidia, hyphae encrusted with crystals, and usually with oil drops inside basidiospores.

**Figure 2.**
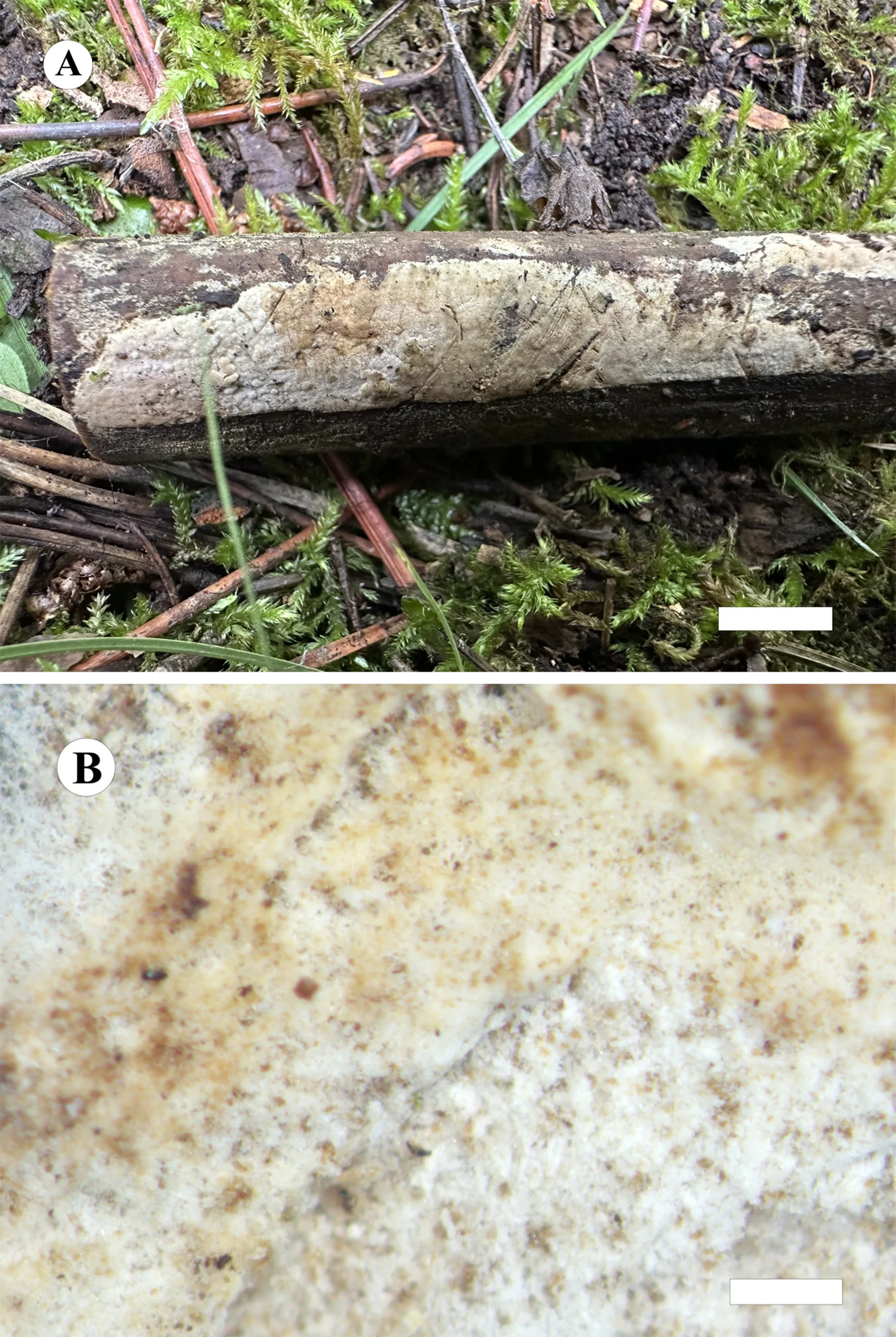
Basidiomata of *Hyphoderma tabuliformis* (holotype, Dai 35238). Scale bars: 1 cm (**A**); 1 mm (**B**).

**Figure 3.**
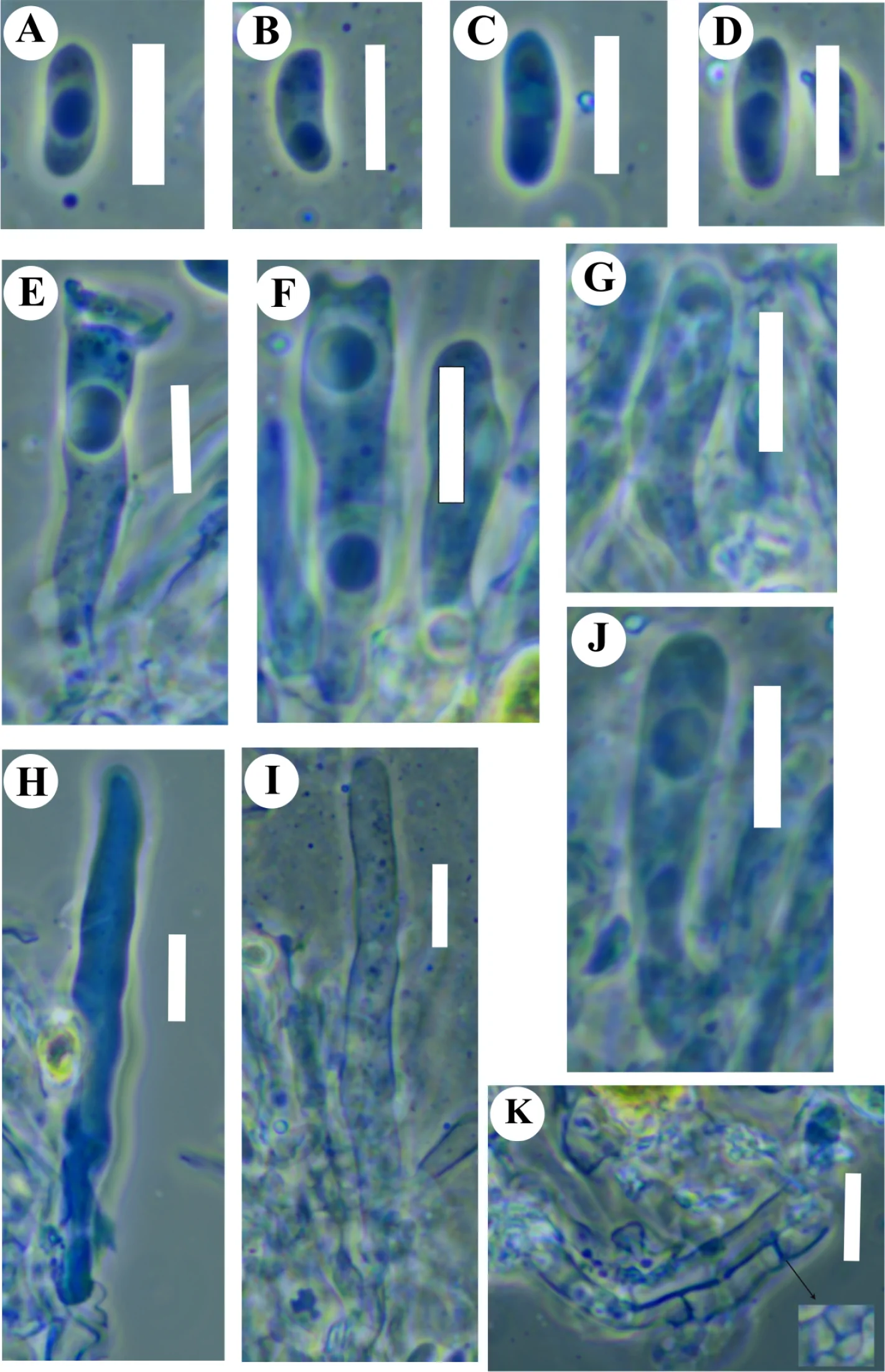
Sections of the hymenium of *Hyphoderma tabuliformis* (holotype, Dai 35238). **A–D**. Basidiospores; **E, F**. Basidia; **G, J**. Basidioles; **H, I**. Cystidia; **K**. Generative hyphae. Scale bars: 10 µm (**A–K**).

**Figure 4.**
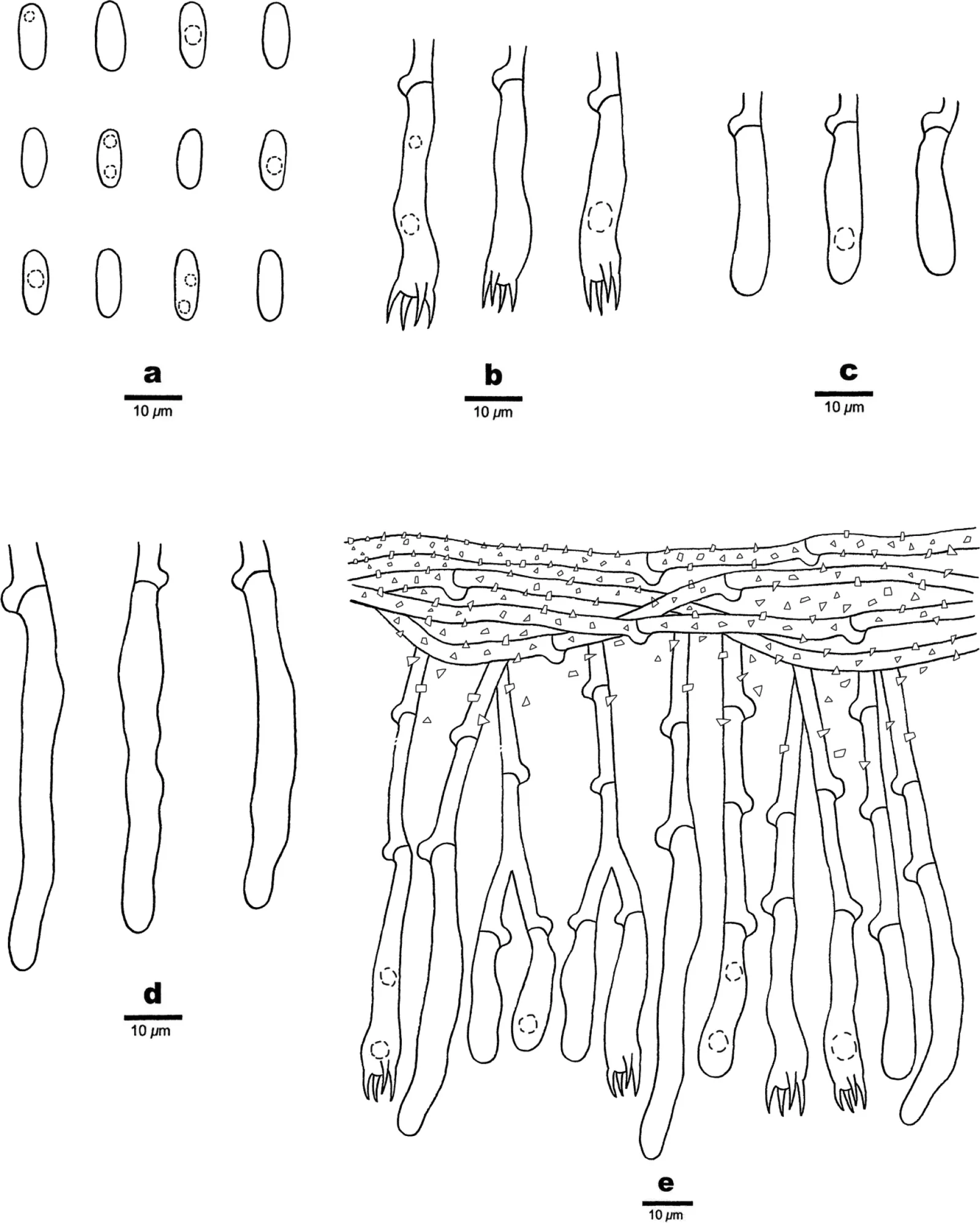
Microscopic structures of *Hyphoderma tabuliformis* (drawn from the holotype). **a**. Basidiospores; **b**. Basidia; **c**. Basidioles; **d**. Cystidia; **e**. Section of basidioma.

##### Holotype.

China • Qinghai Province, Huzhu County, Beishan Forest Park, Langshidang, GPS coordinates 36°53'N, 102°21'E, altitude 2,900 m asl., on a fallen branch of *Pinus tabuliformis*, 10 August 2025, Dai 35238 (holotype BJFC056499).

##### Etymology.

*Tabuliformis* (Lat.): refers to the species growing on *Pinus tabuliformis*.

##### Description.

***Basidiomata***. Annual, resupinate, adnate, without odor and taste, membranaceous to ceraceous when fresh, and up to 7 cm long, 1.6 cm wide, and 100 µm thick at the center. Hymenial surface arachnoid, white to cream when fresh, and pinkish buff when dry. Sterile margin indeterminate, white to cream.

***Subicular hyphae***. Monomitic, generative hyphae with clamp connections, thin-walled, more or less interwoven, encrusted with crystals, 3.0–4.5 µm in diam., IKI–, CB–; tissues unchanged in KOH.

***Cystidia***. Clavate, smooth, thin-walled, 48–68 × 4.8–6.6 µm.

***Basidia***. Clavate, thin-walled, more or less constricted in the middle, usually with oil drops inside, with four sterigmata and a basal clamp connection, 28–40 × 4.0–5.8 µm; basidioles similar to basidia in shape, but smaller.

***Basidiospores***. Subcylindrical to cylindrical, thin-walled, smooth, usually with oil drops inside, IKI–, CB–, (8.4–)8.7–12.2(–11.9) × (3.5–)3.6–4.2(–4.3) µm, *L* = 10.24 µm, *W* = 3.93 µm, *Q* = 2.57–2.64 (*n* = 60/2).

***Type of rot***. White rot.

##### Additional specimen (paratype) examined.

China • Qinghai Province, Huzhu County, Beishan Forest Park, Langshidang, GPS coordinates 36°53'N, 102°21'E, altitude 2,900 m asl., on a fallen branch of *Pinus tabuliformis*, 10 August 2025, Dai 35239 (BJFC056500).

##### Note.

In the phylogenetic tree (Fig. 1), *Hyphoderma tabuliformis* is closely related to *H. medioburiense*, although the ITS region of *H. tabuliformis* differs from that of *H. medioburiense* by 5.8%. *Hyphoderma medioburiense* differs from *H. tabuliformis* by its wider basidia (7–8.5 vs. 4.0–5.8 µm), longer cystidia (70–105 vs. 48–68 µm), and wider basidiospores (4.5–5 vs. 3.6–4.2 µm; Donk 1957). Morphologically, *H. tabuliformis* is similar to *H. alboarachnum* and *H. prosopidis* in sharing an arachnoid hymenial surface and similar hyphal structure. However, *H. alboarachnum* is distinguished from *H. tabuliformis* by having smaller basidiospores (5–6 × 2–3 vs. 8.7–12.2 × 3.6–4.2 µm) and lacking cystidia (Li et al. 2026). *Hyphoderma prosopidis* differs from *H. tabuliformis* by having wider basidia (8.0–11.0 vs. 4.0–5.8 µm) and wider basidiospores (5.5–8.5 vs. 3.6–4.2 µm; Telleria et al. 2012).

#### Hyphoderma tenuicystidiatum

Taxon classificationFungiPolyporalesHyphodermataceae

X. Li, Yuan Yuan & Y.C. Dai sp. nov.

93473390-34CF-5F37-B745-A29C6E83DD4A

864739

Figs 5, 6, 7

##### Diagnosis.

*Hyphoderma tenuicystidiatum* is distinguished by light vinaceous gray hymenial surface when fresh and cream when dry, cylindrical cystidia, fusoid cystidioles and oil drops inside basidiospores.

**Figure 5.**
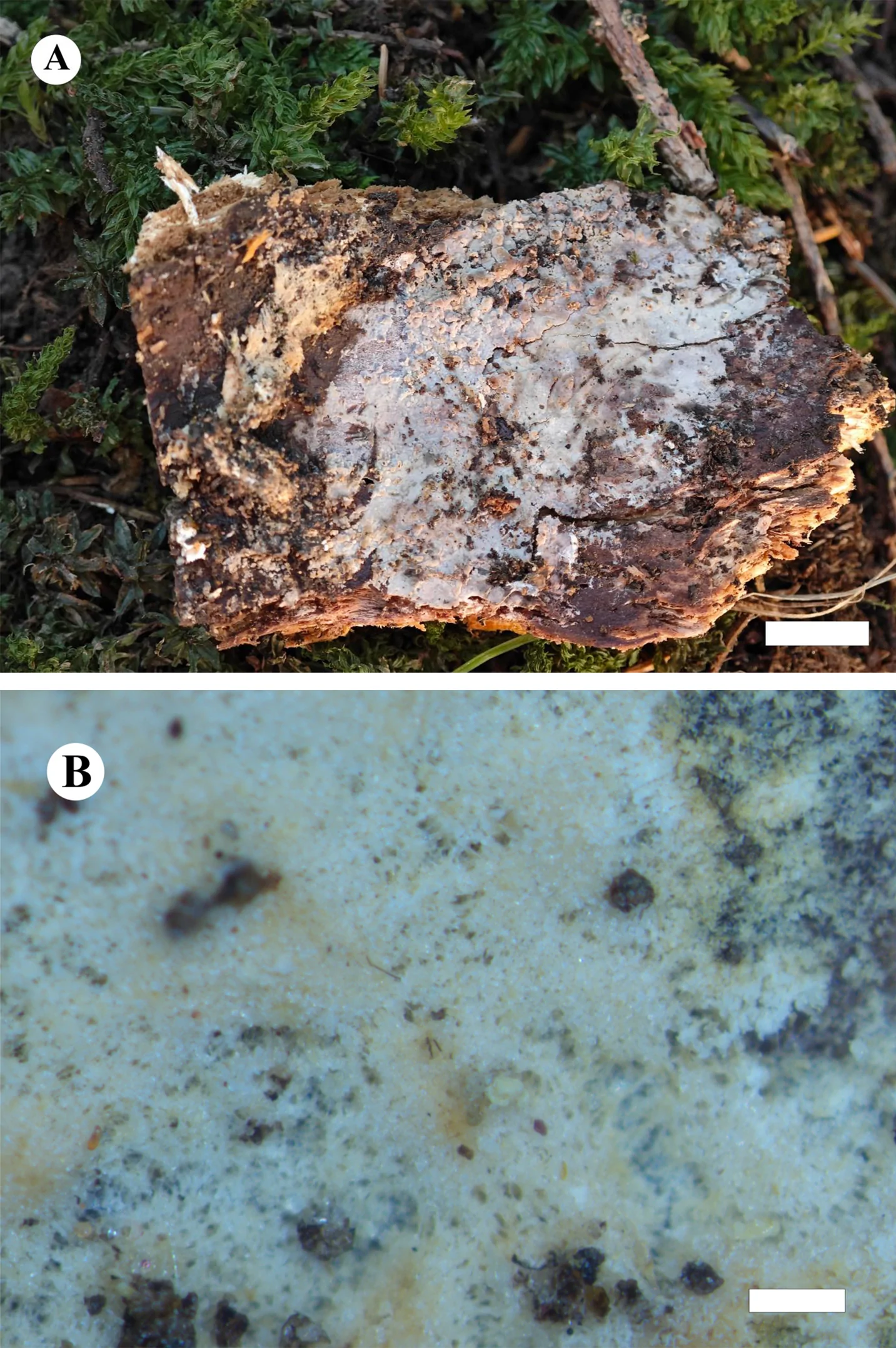
Basidiomata of *Hyphoderma tenuicystidiatum* (holotype, Dai 29223). Scale bars: 1 cm (**A**); 1 mm (**B**).

**Figure 6.**
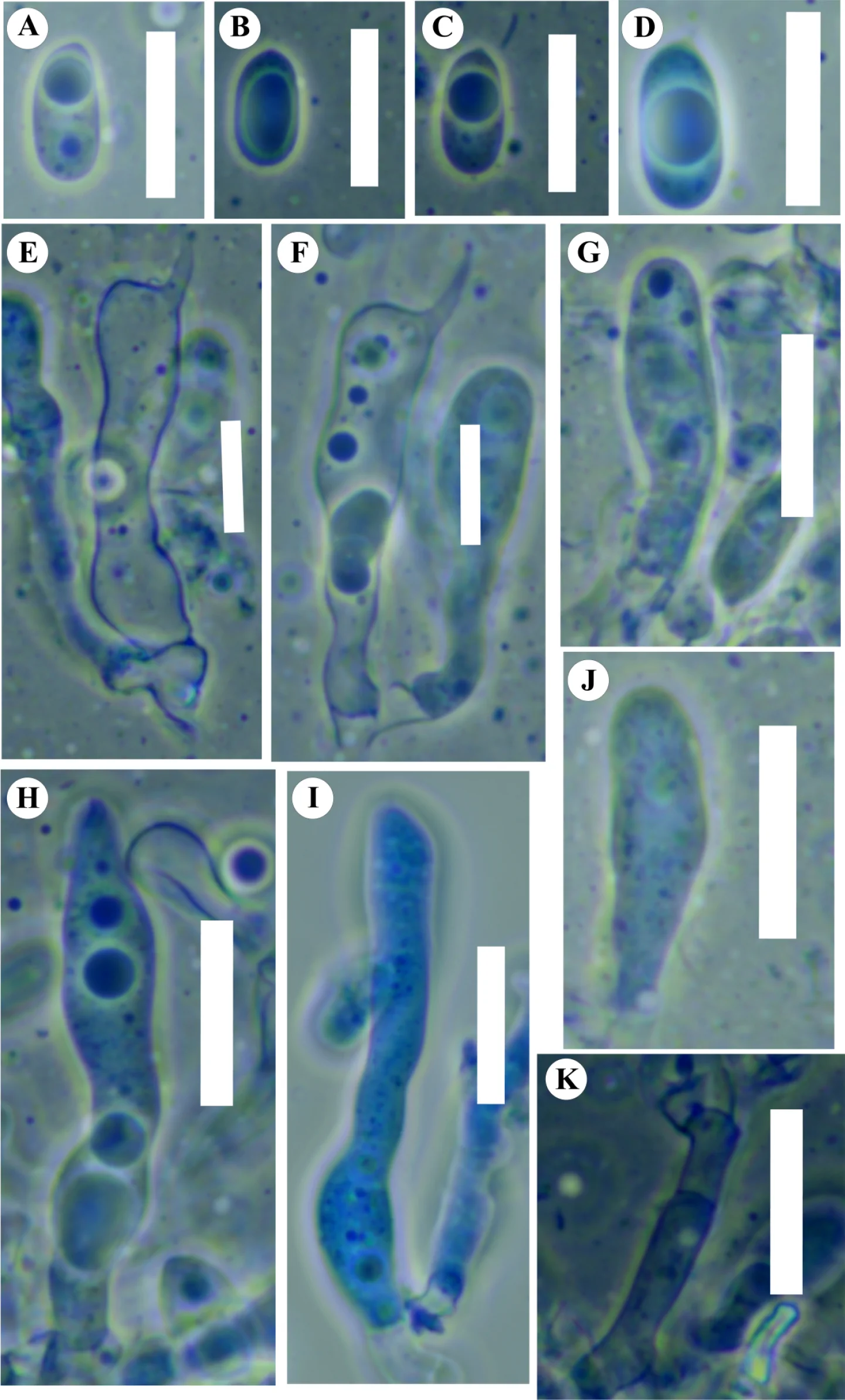
Sections of the hymenium of *Hyphoderma tenuicystidiatum* (holotype, Dai 29223). **A–D**. Basidiospores; **E, F**. Basidia; **G, J**. Basidioles; **H, I**. Cystidia; **K**. Generative hyphae. Scale bars: 10 µm (**A–K**).

**Figure 7.**
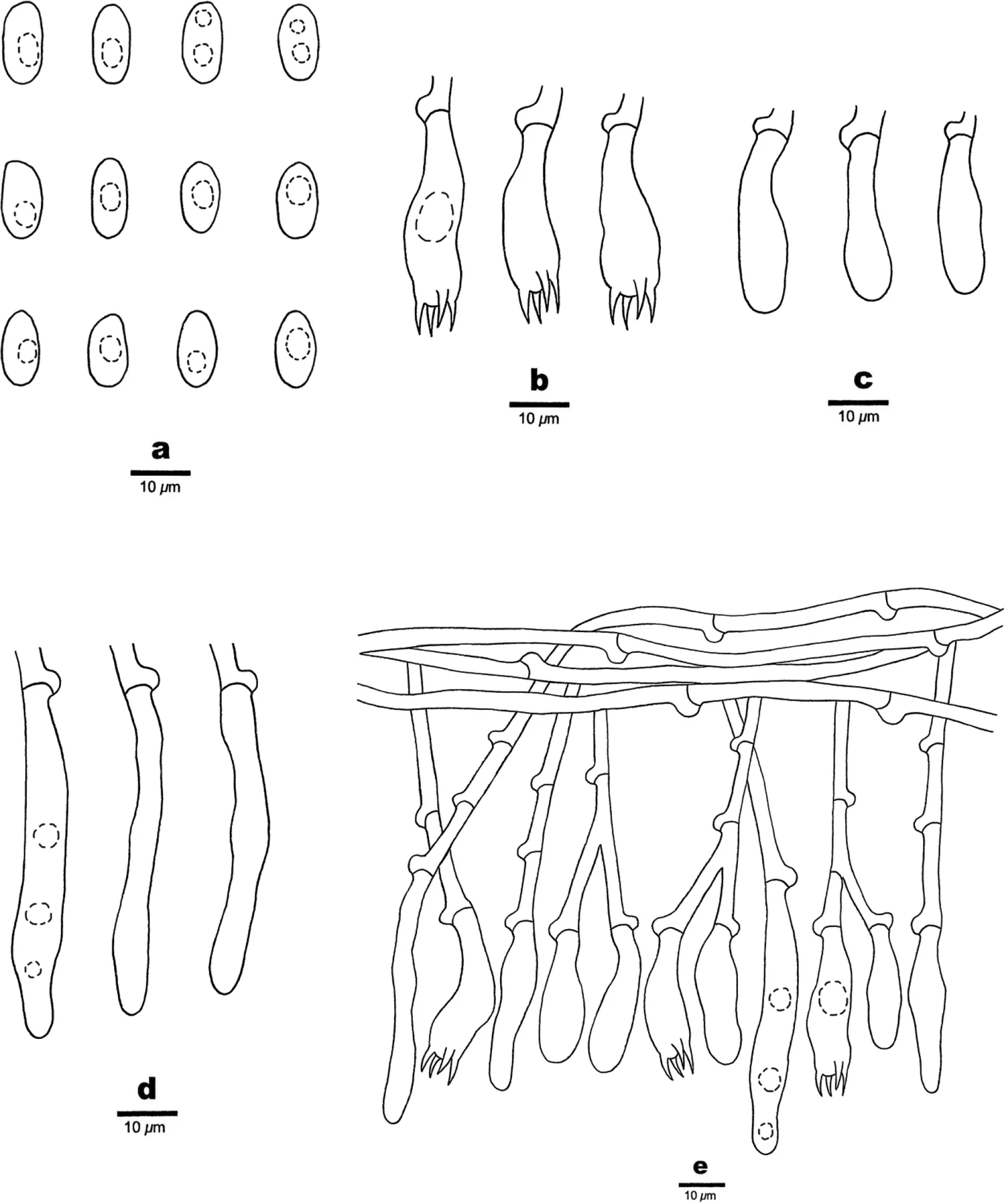
Microscopic structures of *Hyphoderma tenuicystidiatum* (drawn from the holotype). **a**. Basidiospores; **b**. Basidia; **c**. Basidioles; **d**. Cystidia; **e**. Section of basidioma.

##### Holotype.

China • Qinghai Province, Tongren County, Shuangpengxi, GPS coordinates 35°56'N, 102°20'E, altitude 3,068 m asl., on rotten wood of *Picea crassifolia*, 24 July 2024, Dai 29223 (holotype BJFC049482).

##### Etymology.

*Tenuicystidiatum* (Lat.): refers to the species having thin cystidia.

##### Description.

***Basidiomata***. Annual, resupinate, adnate, without odor and taste when fresh, membranaceous, up to 5.0 cm long, 3.5 cm wide, and 100 µm thick. Hymenial surface smooth, light vinaceous gray when fresh, cream when dry. Sterile margin indeterminate, light vinaceous gray.

***Subicular hyphae***. Monomitic, generative hyphae with clamp connections, thin-walled, more or less interwoven, 2.5–3.5 µm diam., CB–, IKI–; tissues unchanged in KOH.

***Cystidia***. Cylindrical, smooth, thin-walled, occasionally with oil drops inside, 40–55 × 4.3–7.7 µm; cystidioles present, fusoid, smooth, thin-walled, 30–38 × 5.4–6.3 µm.

***Basidia***. Clavate, occasionally with oil drops inside, with four sterigmata and a basal clamp connection, 25–38 × 6.9–9.0 µm; basidioles similar to basidia in shape, but smaller.

***Basidiospores***. Subcylindrical to oblong ellipsoid, thin-walled, smooth, with oil drops inside, IKI–, CB–, (9.5–)10.4–14.9(–15.7) × (5.1–)5.2–7.1(–7.2) µm, *L* = 12.30 µm, *W* = 6.12 µm, *Q* = 1.89–2.04 (*n* = 60/2).

***Type of rot***. White rot.

##### Additional specimen (paratype) examined.

China • Qinghai Province, Xunhua County, Mengdatianchi Nature Reserve, GPS coordinates 35°51'N, 102°38'E, altitude 3,000 m asl., on a dead angiosperm tree, 12 October 2024, Dai 31215 (BJFC051474).

##### Notes.

In the phylogenetic tree (Fig. 1), *Hyphoderma tenuicystidiatum* and *H. tabuliformis* formed two well-supported lineages (100% in ML and 1.00 BPP; 100% in ML and 1.00 BPP, respectively). The phylogenetic analyses revealed that *H. tenuicystidiatum* is related to *H. obtusiforme*, but the ITS region of *H. tenuicystidiatum* differs from that of *H. obtusiforme* by 4.4%. *Hyphoderma obtusiforme* is distinguished from *H. tenuicystidiatum* by having wider cystidia (8–10 vs. 4.3–7.7 µm; Eriksson and Ryvarden 1975). Morphologically, *H. tenuicystidiatum* is highly similar to *H. microporoides*, *H. fissuratum*, and *H. transiens* in having clavate basidia, similar hyphal structure, and a smooth hymenial surface. However, *H. microporoides* differs from *H. tenuicystidiatum* by having smaller basidiospores (8.5–10 × 2.5–3.5 vs. 10.4–14.9 × 5.2–7.1 µm) and capitate cystidia (Guan and Zhao 2021). *Hyphoderma fissuratum* is distinguished from *H. tenuicystidiatum* by having smaller basidiospores (8.5–10.3 × 3–4 vs. 10.4–14.9 × 5.2–7.1 µm) and lacking cystidia (Ma et al. 2021). *Hyphoderma transiens* differs from *H. tenuicystidiatum* by having narrower basidiospores (3.5–4 vs. 5.2–7.1 µm; Parmasto 1968).

## Discussion

In this study, morphological and phylogenetic analyses were conducted on *Hyphoderma* specimens collected from Qinghai Province, and two new species were identified and described (Fig. 1; Table 1), raising the total number of *Hyphoderma* species known from China from 45 to 47 (Yang et al. 2023; Li et al. 2026). Because the molecular phylogenetic analyses included only a subset of the currently accepted species of *Hyphoderma*, phylogenetic placement alone cannot exclude the possibility that the newly collected specimens correspond to described species for which molecular data are unavailable. To address this issue, the two new species were compared with morphologically similar accepted species, especially those lacking molecular data, as well as those represented in the phylogenetic analyses (Table 2). The morphological characters compared included basidiomata morphology; cystidia type, shape, and size; basidia shape and size; and basidiospore shape and size. Therefore, although some accepted *Hyphoderma* species still lack molecular data, the recognition of the new species is supported by an integrated combination of molecular phylogenetic position, morphological diagnosis, comparison with the closest phylogenetic relatives, and comparison with morphologically similar species.

**Table 2.** Morphological comparison of the new species with similar taxa of the genus *Hyphoderma*.

**Species name**	**Basidiomata**	**Hyphal system**	**Cystidia**	**Basidia**	**Basidiospores**
*Hyphoderma acutatum*	Hymenial surface smooth, whitish to ochraceous	Monomitic, thin-walled, generative hyphae with clamp connections	Subulate, thin-walled, 100–170 × 15–20 µm	Suburniform, 25–30 × 5–6 µm, four sterigmata, a basal clamp connection	Ellipsoid, 7–7.5(–8–9) × 4–4.5(–5) µm
*H. alboarachnum*	Hymenial surface arachnoid, white to cream	Monomitic, thin-walled, generative hyphae with clamp connections	Absent	Cylindrical, 23.5–30 × 3.5–4.5 µm, four sterigmata, a basal clamp connection	Oblong–ellipsoid to cylindrical; 5–6 × 2–3 µm.
*H. ayresii*	Hymenial surface smooth, whitish to pale pinkish buff	Monomitic, thin- to slightly thick-walled, generative hyphae with clamp connections	Subulate, thick-walled, encrusted, 60–160 × 15–30 µm	Subutriform to subclavate, 37–47 × 10–12.5 µm, four sterigmata, a basal clamp connection	Ellipsoid, 11.5–13.5 × 7–8 μm
*H. bicystidiatum*	Hymenial surface smooth, yellowish white to pale yellow	Monomitic, thin-walled, generative hyphae with clamp connections	1) tubular, thin-walled, 82.0–122.0 × 12.3–13.8 µm. 2) clavate to subclavate, thick-walled, 69.0–110.0 × 11.0–13.8 µm	Clavate to subclavate, 13.6–22.3 × 6.2–7.5 µm, four sterigmata, a basal clamp connection	subellipsoid to suballantoid, 6.2–8.8 × 3.0–4.1 µm
*H. clavatum*	Hymenial surface smooth or slightly tuberculate, grayish clay	Monomitic, thin-walled, generative hyphae with clamp connections	Clavate or apically globose, thin-walled or apically slightly thick-walled, 30–60 µm long, 10–15 µm diam for the globose parts	Subclavate, 30–40 × 6.5–8 µm, four sterigmata, a basal clamp connection	Cylindrical, 10–13 × 4.2–5.2 µm
*H. fissuratum*	Hymenial surface smooth, cream	Monomitic, thin-walled, generative hyphae with clamp connections	Absent	Narrowly clavate, 24–28 × 4–5.5 µm, four sterigmata, a basal clamp connection	Cylindrical, 8.5–10.2 × 3–4 µm
*H. galactinum*	Hymenial surface smooth to tuberculate	Monomitic, thin-walled, generative hyphae with clamp connections	Cylindrical, often more or less moniliform with several constrictions, thin to slightly thick-walled, 40–80 × 6–10 µm.	Subclavate to subventricose, 25–35 × 7–8 μm, four sterigmata, a basal clamp connection	Cylindrical to suballantoid, 13–16 × 4–4.5 μm
*H. hjortstamii*	Hymenial surface smooth, yellowish white to pale yellow	Monomitic, thin-walled, generative hyphae with clamp connections	Subcylindrical, thin-walled, 40–55 × 5.6–6.8 µm	Clavate, 17–26 × 5–6.8 µm, four sterigmata, a basal clamp connection	Suballantoid to allantoid, 8–11 × 3.1–4.4 µm
*H. incrustatum*	Hymenial surface smooth to tuberculate, pale orange to greyish orange to greyish red	Monomitic, thin-walled, generative hyphae with clamp connections	1) tubular, thin-walled, encrusted, 76–90 × 7–8 µm. 2) capitate, encrusted, 33–43 × 4.6–11.7 µm	Clavate, 27–39 × 6.2–8.7 μm, four sterigmata, a basal clamp connection	Ellipsoid to suballantoid, 10.8–16 × 4.3–5.2 μm
*H. lunasporum*	Hymenial surface smooth, white to cream	Monomitic, thin-walled, generative hyphae with clamp connections	Cylindrical to subclavate, thin- to slightly thick-walled, 30–60 × 5–8 µm	Subclavate to suburniform, 20–30 × 5–7 μm, four sterigmata, a basal clamp connection	Allantoid to lunate, 8–12 × 3–4.5 μm
*H. malenconii*	Hymenial surface smooth to tuberculate, ivory yellow to light buff to pale olive–buff	Monomitic, thin- to slightly thick-walled, generative hyphae with clamp connections	Cylindrical, thin-walled, 35–90 × 7–12 µm	Utriform to subutriform, 30–50 × 7–9 µm, four sterigmata, a basal clamp connection	Cylindrical, 12–15.5 × 5–6 μm
*H. microcystidium*	Hymenial surface smooth, whitish	Monomitic, thin- to slightly thick-walled, generative hyphae with clamp connections	1) Lamprocystidia, slightly thick-walled, encrusted, 17–32 × 7–10.5 µm. 2) Leptocystidia, cylindrical, 20–65 × 8–13 µm	Subutriform, 18–25 × 6.5–7.5 µm, four sterigmata, a basal clamp connection	Narrowly ellipsoid, 10–12.5 × 4–4.8 μm
*H. microporoides*	Hymenial surface smooth, cream to pale buff	Monomitic, thin-walled, generative hyphae with clamp connections	Capitate halocystidia, thin-walled, 18–51 × 4.5–7 µm	Clavate, 18.5–29.5 × 5–7 µm, four sterigmata, a basal clamp connection	Cylindrical to allantoid, 8.5–10 × 2.5–3.5 µm
*H. rimulosum*	Hymenial surface smooth, white to ivory yellow	Monomitic, thin-walled, generative hyphae with clamp connections	Cylindrical, slightly thick-walled, usually encrusted throughout, 30–55 × 4–6 µm	Subclavate, 22–32 × 4.5–6 µm, four sterigmata, a basal clamp connection	Ellipsoid, 6–7 × 3.9–4.1 μm
*H. subclavatum*	Hymenial surface smooth, clay–yellow	Monomitic, thin- to slightly thick-walled, generative hyphae with clamp connections	Clavate, thin-walled, 40–70 × 8–11 µm, apically often with one to several secondary septa.	Suburniform, 40–55 × 7–8 µm, four sterigmata, a basal clamp connection	Cylindrical, 10–12 × 4.2–5.3 µm
*H. subglobosum*	Hymenial surface smooth, grayish yellow to grayish orange	Monomitic, thin-walled, generative hyphae with clamp connections	Clavate, thin-walled, 50.0–69.0 × 8.0–11.8 µm	Clavate to subclavate, 22.0–34.0 × 6.8–10.0 µm with oily contents, four sterigmata, a basal clamp connection	Subglobose to broadly ellipsoid, 11.8–13.1 × 8.0–10.0 µm
***H. tabuliformis***	Hymenial surface arachnoid, white to cream	Monomitic, thin-walled, generative hyphae with clamp connections	Clavate, thin-walled, 48–68 × 4.8–6.6 µm	Clavate, 28–40 × 4.0–5.8 µm, four sterigmata, a basal clamp connection	Subcylindrical to cylindrical, 8.7–12.2 × 3.6–4.2 µm
***H. tenuicystidiatum***	Hymenial surface smooth, light vinaceous gray	Monomitic, thin-walled, generative hyphae with clamp connections	Cylindrical, thin-walled, 40–55 × 4.3–7.7 µm	Clavate, 25–38 × 6.9–9.0 µm, four sterigmata, a basal clamp connection	Subcylindrical to oblong ellipsoid, 10.4–14.9 × 5.2–7.1 µm
*H. transiens*	Hymenial surface odontioid, whitish, capucine buff to light ochraceous buff	Monomitic, thin-walled, generative hyphae with clamp connections	Subcylindrical to cylindrical, thin-walled, 63.0–76.0 × 9.4–11.8 μm	Clavate to subclavate, 20.0–32.0 × 6.2–7.6 μm, four sterigmata, a basal clamp connection	Ellipsoid to suballantoid, 9.6–10.6 × 3.4–4.2 μm

In terms of ecology and host associations, *Hyphoderma* is an important genus of wood-decaying fungi, widely distributed worldwide and playing a key role in forest carbon cycling (Dai et al. 2021; Zhou et al. 2023). Most *Hyphoderma* species grow on hardwoods, whereas a few occur on coniferous wood (Dai et al. 2004b). The two new species differ in host use: *H. tenuicystidiatum* was found on rotten wood of *Picea* and dead angiosperm trees, indicating a relatively broad host range, whereas *H. tabuliformis* was found only on fallen branches of *Pinus tabuliformis*, appearing to be more specialized. This suggests that different species within the genus may have distinct host preferences and ecological adaptation strategies, potentially reflecting differences in lignin-degrading capacity or niche partitioning, although most species are not strictly selective in their wood substrates (Dai 2010; Yurchenko and Wu 2015; He et al. 2019). Because sampling was restricted to a limited area in Qinghai, additional *Hyphoderma* species may remain to be discovered in under-surveyed areas, such as the margins of the Qinghai–Tibet Plateau and the boreal coniferous forest zones of northeastern China. In addition, the present study relied solely on ITS and nLSU sequences, mainly because the vast majority of *Hyphoderma* species currently lack RPB1, RPB2, and TEF1 sequences in public databases, and incorporating these genes would substantially reduce taxonomic coverage. Therefore, future studies should expand sampling across broader geographic and habitat ranges, generate and deposit multilocus sequences of *Hyphoderma* in public databases, and incorporate these data into future analyses to further investigate the diversity, taxonomy, and evolution of *Hyphoderma*.

### Key to species of *Hyphoderma* from China

**Table:** 

1	Cystidia absent	**2**
–	Cystidia present	**5**
2	Hymenial surface arachnoid	***H. alboarachnum***
–	Hymenial surface smooth	**3**
3	Hymenial surface cream when fresh	***H. fissuratum***
–	Hymenial surface white when fresh	**4**
4	Generative hyphae with clamp connection	***H. weishanense***
–	Generative hyphae simple with simple-septa	***H. yingjiangense***
5	One type of cystidia present	**6**
–	Two types of cystidia present	**28**
6	Cystidia encrusted with crystals	**7**
–	Cystidia not encrusted with crystals	**12**
7	Basidiospores > 7.5 µm long	**8**
–	Basidiospores < 7.5 µm long	**11**
8	Cystidia > 60 µm long	**9**
–	Cystidia < 60 µm long	***H. fulgens***
9	Cystidia thick-walled	**10**
–	Cystidia thin-walled	***H. yunnanense***
10	Cystidia subulate	***H. macrosporum***
–	Cystidia fusiform	***H. mopanshanense***
11	Cystidia cylindrical	***H. rimulosum***
–	Cystidia tubular	***H. puerense***
12	Basidiospores > 6.5 µm long	**13**
–	Basidiospores < 6.5 µm long	**27**
13	Basidia 2-sterigmata	***H. fragilissimum***
–	Basidia 4-sterigmata	**14**
14	Halocystidia absent	**15**
–	Halocystidia present	**25**
15	Septate cystidia absent	**16**
–	Septate cystidia present	**24**
16	Cheilocystidia absent	**17**
–	Cheilocystidia present	***H. marginatum***
17	Hymenial surface grandinioid	***H. grandineum***
–	Hymenial surface smooth	**18**
18	Basidia > 40 µm long	***H. subclavatum***
–	Basidia < 40 µm long	**19**
19	Basidiospores > 6 µm wide	**20**
–	Basidiospores < 6 µm wide	***H. tabuliformis***
20	Basidiospores > 10 µm long	**21**
–	Basidiospores < 10 µm long	**23**
21	Basidiomata membranaceous	**22**
–	Basidiomata subceraceous	***H. hjortstamii***
22	Basidiospores cylindrical	***H. clavatum***
–	Basidiospores subcylindrical to oblong ellipsoid	***H. tenuicystidiatum***
23	Cystidia cylindrical	***H. litschaueri***
–	Cystidia moniliform	***H. asianum***
24	Hymenial surface cracked	***H. subsetigerum***
–	Hymenial surface not cracked	***H. subpraetermissum***
25	Basidia clavate	**26**
–	Basidia barrelled	***H. qujingense***
26	Basidiospores > 8.5 µm long	***H. microporoides***
–	Basidiospores < 8.5 µm long	***H. punctatum***
27	Basidia clamped at base	***H. sordidum***
–	Basidia simple-septate at base	***H. bambusinum***
28	Septate cystidia absent	**29**
–	Septate cystidia present	**32**
29	Basidiospores > 9 µm long	**30**
–	Basidiospores < 9 µm long	***H. laceratum***
30	Basidia clamped at base	**31**
–	Basidia simple-septate at base	***H. cinereofuscum***
31	Basidiospores allantoid	***H. crystallinum***
–	Basidiospores cylindrical	***H. nitidum***
32	Basidia 2-sterigmata	***H. pinicola***
–	Basidia 4-sterigmata	**33**
33	Hymenial surface grandinioid	***H. setigerum***
–	Hymenial surface smooth	***H. guangdongense***

## Supplementary Material

XML Treatment for Hyphoderma tabuliformis

XML Treatment for Hyphoderma tenuicystidiatum

## Additional information

### Artificial Intelligence (AI) use

The authors accept full responsibility for the content of the manuscript, including the disclosure of any use of AI.

No AI tools were used in the preparation of this manuscript.

### Author ORCIDs

X. Li https://orcid.org/0009-0001-1625-8589

Y. Cui https://orcid.org/0009-0000-8706-6771

Y. Dai https://orcid.org/0000-0002-6523-0320

Y. Yuan https://orcid.org/0000-0001-6674-9848

Y. Wu https://orcid.org/0000-0003-1295-4015

### Data availability

All of the data that support the findings of this study are available in the main text.
